# The Interplay of Angiogenesis and Osteogenesis in Non‐Stabilized Incomplete Tibial Fractures: A Temporal Study in Rats

**DOI:** 10.1002/jor.70006

**Published:** 2025-06-20

**Authors:** Kyung Wook Kim, Andrew Reyes Padalhin, Hyun Seok Ryu, Celine Abueva, So Young Park, Jun Sang Bae, Seung Hyeon Yoo, Hwee Hyon Seo, Phil‐Sang Chung, Hyun Sik Gong, Seung Hoon Woo

**Affiliations:** ^1^ Department of Orthopaedic Surgery, Dankook University College of Medicine Dankook University Hospital Cheonan Republic of Korea; ^2^ Department of Orthopedic Surgery, Seoul National University College of Medicine Seoul National University Bundang Hospital Seongnam Republic of Korea; ^3^ Dankook Institute of Medicine and Optics Dankook University Cheonan Republic of Korea; ^4^ Beckman Laser Institute Korea Dankook University Cheonan Republic of Korea; ^5^ Medical Laser Research Center Dankook University Cheonan Republic of Korea; ^6^ School of Medical Laser Dankook University Graduate School Dankook University Cheonan Republic of Korea; ^7^ Department of Otorhinolaryngology‐Head and Neck Surgery, Dankook University College of Medicine Dankook University Hospital Cheonan Republic of Korea

**Keywords:** angiogenesis‐osteogenesis coupling, non‐stabilized long bone fracture, vascularization

## Abstract

Prior research on angiogenesis and osteogenesis during bone fracture healing has primarily focused on stabilized models, often within controlled environments. However, the dynamic interplay of these processes in the context of long bone fractures without scaffolds or external factors remains poorly understood. This study investigated the temporal dynamics of angiogenesis and osteogenesis in a non‐stabilized incomplete transverse tibia bone fracture model. Multi‐modal observation approach was carried out using micro‐CT analysis of bone mineral density, histological assessment of fracture healing, and confocal microscopy for visualization and co‐localization of key angiogenic (CD31, endomucin) and osteogenic (collagen 1, osteocalcin, PDGFRB) markers. Our findings revealed a dynamic and interdependent relationship between these processes. H type blood vessels persisted throughout the healing process, and significant correlations were observed between the expression of angiogenic and osteogenic markers. The role of hypoxia, a critical regulator of both processes, was investigated by analyzing HIF1‐α expression. Increased expression of SLIT3, a guidance molecule, was also observed at later stages of healing, suggesting its potential involvement in vascular remodeling. These findings provide crucial insights into the molecular mechanisms underlying bone fracture repair in the absence of additional supporting external elements such as scaffolds or stabilizers. By elucidating the temporal dynamics of these key processes, we identify potential targets for therapeutic interventions aimed at accelerating and optimizing bone regeneration. Consideration of these interactions can lead to the development of effective clinical strategies for enhancing bone healing in non‐stabilized incomplete fractures.

## Graphical abstract

This study explores the interplay between angiogenesis and osteogenesis during bone healing in a non‐stabilized transverse fracture model. Employing micro‐CT, histology, and confocal microscopy, it identifies key temporal dynamics and molecular markers such as H type blood vessels, HIF1‐α, PDGFRB, and SLIT3. The findings highlight the persistent coupling of vascular and bone tissue repair processes, offering insights into therapeutic targets to accelerate fracture healing. The graphical abstract visually integrates stages of repair, marker expression, and their interdependence.

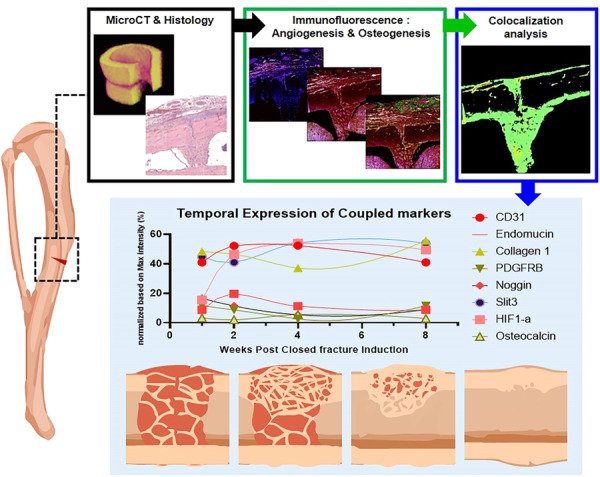

**Abbreviations**
BMP: Bone morphogenetic protein
HIF1‐α: Hypoxia‐inducible factor 1‐alpha
H&E: Hematoxylin and eosin
IF: Immunofluorescence
Micro‐CT: Micro computed Tomography
PDGFRB: Platelet‐derived growth factor receptor beta
SLIT3: Slit Guidance Ligand 3
VEGF: Vascular endothelial growth factor

## 1 Introduction

Bone tissue consists of a mineralized matrix encased by a membranous periosteal layer and is richly vascularized. Its blood supply includes nutrient arteries that penetrate the diaphysis, periosteal vessels that nourish the outer layers, and metaphyseal and epiphyseal vessels located near the ends of long bones. The general structure of blood vessels involves an endothelial lining and a basement membrane, with larger vessels benefiting from the support of pericytes and smooth muscle cells [1, 2, 3]. However, the bone's unique demands necessitate specialized vascularization. Consequently, the bone is supplied by primarily two types of blood vessels: L type along the diaphysis and H type in the growth plate and metaphysis. Considering the critical role of vascularization in skeletal repair, it's significant that fracture healing, a complex biological process involving the coordinated action of numerous cellular and molecular components, heavily depends on H type blood vessels, a specialized vascular network within the skeletal system [4, 5, 6]. Furthermore, these H type blood vessels, also found in the bone growth plates and metaphysis, are closely associated with osteogenic activity [7, 8]. They are characterized by high expression of endomucin and CD31 [4, 5, 9]. These vessels form in the cancellous bone area during long bone development to provide adequate nutritional support for cells near the growth plate. This nutrient supply ensures the proper development and maintenance of bone tissue, allowing for a larger surface area for nutrient and oxygen exchange [4, 5, 6, 10].

Beyond nutrient delivery, H type blood vessels also support hematopoiesis by creating a conducive environment for hematopoietic stem cells within the highly vascularized bone marrow [4, 5, 9]. These vessels provide physical support and deliver essential growth factors and signaling molecules, promoting the proliferation, differentiation, and maturation of blood cells. Additionally, they regulate the immune response within the bone marrow, ensuring effective immune cell trafficking. In the context of bone formation, H type blood vessels actively participate in osteogenesis by providing a scaffold for the deposition of mineralized matrix and regulating the activity of bone‐forming cells such as osteoblasts [7, 8]. They influence osteoblast activity through the delivery of growth factors and signaling molecules, facilitating the balance between bone formation and resorption.

Rats are widely used in bone regeneration studies because they share biological similarities with humans, have a fast healing process, and their bones are structurally comparable to human bones. They are cost‐effective and easier to manage than larger animals, and established models allow for consistent studies on bone defects [11, 12, 13, 14, 15, 16]. Although previous research has characterized H type blood vessels, these studies fall short of observing the said phenomena under non‐stabilized and non‐scaffold‐based studies. Therefore, exploring the role and interactions of angiogenic and osteogenic markers in bone fractures can lead to several intriguing research questions. Studying non‐stabilized incomplete fractures, which mimic clinical scenarios, offers a unique opportunity to understand the body's natural healing mechanisms, the role of controlled micromotion in bone repair and angiogenesis, early healing events, and potentially identify novel therapeutic targets when rigid fixation is not ideal. Understanding the temporal dynamics involves examining how the expression levels of angiogenic markers (e.g., CD31, endomucin) [17, 18, 19, 20] and osteogenic markers (e.g., osteocalcin, platelet‐derived growth factor receptor beta (PDGFRB)) change over the course of fracture healing [21, 22, 23, 24], and identifying the specific time points at which these markers peak and how these peaks correlate with different stages of bone repair. Additionally, the role of hypoxia, indicated by markers such as Hypoxia‐inducible factor 1‐alpha (HIF1‐α) [25, 26, 27], in regulating angiogenesis and osteogenesis during fracture healing, and how the hypoxic environment affects the expression and function of both angiogenic and osteogenic markers, is another area of interest. More so, understanding the localization of developmental and guidance markers such as noggin [28, 29, 30] and SLIT3 [31, 32, 33], linked with the coupling of angiogenesis and osteogenesis can also provide additional information on possible routes of novel treatments for bone regeneration. The authors hypothesize that there is a dynamic and interdependent relationship between angiogenesis and osteogenesis which could be observed better in a non‐stabilized incomplete transverse tibia bone fracture animal model. This study explores the temporal dynamics of angiogenesis (blood vessel formation) and osteogenesis (bone formation) during the healing of non‐stabilized incomplete transverse tibia bone fractures in rats. Doing so will help identify potential targets for therapeutic interventions aimed at accelerating and optimizing bone regeneration.

## 2 Methods

### 2.1 Non‐Stabilized Incomplete Transverse Bone Fracture Animal Modeling

All procedures for this experiment were evaluated, approved, and conducted according to the rules and regulations set by the Institutional Animal Care and Use Committee of Dankook University (DKU‐22‐027). Male Sprague‐Dawley (SD) rats, aged 6 weeks, were obtained from Orient Bio (Songnam, South Korea). Animals were provided with ad libitum food and water and acclimatized for 1 week in a climate‐ controlled animal room with 12‐h light‐ dark cycle before induction of the incomplete transverse tibial fracture. The 24 rats were randomly divided into four groups, with six rats per group, accounting for 1, 2, 4, and 8 weeks post‐fracture induction observation points. Surgical procedures were performed in a sterile operating room with the animal under anesthesia using isoflurane inhalation. Isoflurane was used considering its efficacy and wide adaptation for bone surgical procedures for animal experiments [34, 35, 36]. Upon full anesthetic induction, the animal was positioned supine on the operating table and the fur was shaved from the right leg shank along the surface of the tibial bone. The exposed skin was then cleaned with 70% ethanol and disinfected with povidone‐iodine in preparation for skin‐level incision. To access the tibia bone, a longitudinal incision measuring 1.5–2.0 cm in length was made approximately 1.5 cm below the knee joint. The overlaying periosteal layer was cut and carefully retracted laterally to fully reveal the tibial bone surface. An incomplete transverse fracture was simulated by creating a 2 mm deep cut along the tibial diaphysis using a circular saw disk while being constantly irrigated with sterile saline solution. Upon completing the cut, the retracted periosteum was repositioned over the bone surface and secured with sutures. The skin‐level incision was also closed using sutures. The operated leg was secured to the side of the animal with a minimal amount of casting tape, which was removed after 3 days. The animal was then returned to its home cage to recover fully. Postoperatively, the animals were regularly monitored for signs of severe distress. Postoperatively, the animals were regularly monitored for signs of severe distress.

### 2.2 Micro‐CT Analyses

At the indicated time point (1, 2, 4, 8 weeks post incomplete transverse fracture induction), the rats were killed by carbon dioxide inhalation. The legs were then isolated and fixed with 10% formalin for 3–4 days. Surrounding soft tissues were removed and specimens were pat‐dried and wrapped with parafilm in preparation for microcomputed tomography (micro‐CT) scanning. Tissue samples were scanned with a Skyscan 1176 micro‐CT scanner (Bruker micro‐CT, Belgium) with the device set at 65 kV, with a source current of 385 μA through a 1.0 mm aluminum filter. Submicron scans were taken 360 degrees with a 1.0‐degree rotational step and 400 ms exposure time. Data sets were then reconstructed using Nrecon software (Bruker micro‐CT, Belgium) and rotated using Data Viewer (Bruker micro‐CT, Belgium). We then analyzed the diaphyseal section measuring 3.0 mm × 3.0 mm × 4.0 mm of the rotated datasets using CTAn software (Bruker micro‐CT, Belgium) to determine the volumetric bone mineral density of cortical and trabecular bone. For visualization, three‐dimensional reconstructions of the micro‐CT data were generated from representative data sets using CTVox software (Bruker micro‐CT, Belgium). Samples that resulted in complete irregular malunion due to total breakage were no longer included in further analyses.

### 2.3 Tissue Section Staining and Analyses

#### 2.3.1 Hematoxylin and Eosin Staining for Visualization of Regenerated Bone

The morphological structure of the regenerated bone tissue was observed by preparing stained tissue sections. Extracted tibia bone tissue samples underwent a decalcification process using a 10% v/v EDTA solution at room temperature for a week, with the solution replaced every other day to ensure thorough removal of minerals. Subsequently, the decalcified tissues were processed through a series of alcohol solutions to dehydrate them, followed by immersion in xylene to clear them. These tissues were then infiltrated with paraffin using an automated Tissue‐Tek VIP®5 Jr. processing machine (Sakura Finetek, USA). The paraffin‐infiltrated tissue samples were embedded in paraffin blocks, from which 5.0 µm sections were cut using a Leica RM2135 microtome (Leica, Germany). These sections were then stained with hematoxylin and eosin (H&E). The stained tissue sections were imaged using an Olympus BX53 light microscope equipped with cellSens imaging software (Build 18987).

#### 2.3.2 Immunofluorescence for Angiogenesis and Osteogenesis Coupling

Immunofluorescence (IF) staining was conducted to examine the expression of tissue markers associated with both H type blood vessel formation and bone regeneration. For antigen retrieval of the deparaffinized and rehydrated tissue sections, a heat‐induced antigen retrieval method was employed using citrate buffer (10 mM, pH 6.0). This process facilitated the exposure of epitopes for subsequent antibody binding. The tissue sections were then blocked with 3% bovine serum albumin (BSA) to minimize nonspecific antibody binding. Individual slides were incubated with paired primary antibodies specifically chosen to investigate either angiogenesis or osteogenesis. Table 1 provides a detailed list of the primary and secondary antibodies utilized in this study. Immunofluorescent stained tissue sections mounted with Vectashield (H‐1200, Vector Laboratories, Burlingame) containing DAPI for counter‐staining cell nuclei. Micrographs were then taken via z‐tacked multi‐area time‐lapse (MATL) in a 3 × 3 area grid with subsequent stitching using the FV‐3000 software (Olympus, Tokyo, Japan). Images were then analyzed for colocalization and co‐relation of stained tissue markers for angiogenesis and osteogenesis using FIJI (ImageJ with plugins). Mean fluorescence intensities for green and red channels of the confocal images were measured using conventional mean gray value measurements with specifications indicated through regions of interest. Colocalization analysis was used to determine the colocalized expression of paired markers for angiogenesis and osteogenesis. This was accomplished by using the JACoP plugin [37]. To visualize colocalization, a color map was then generated using the Colocalization Color Map plugin [38].

**Table 1:** Primary and secondary antibodies used for observing angiogenesis and osteogenesis.

Primary antibody		Catalogue number/company
Angiogenesis	Goat anti‐ CD31/PECAM‐1	(AF3628, R&D Systems)
	Rabbit anti‐Endomucin	(PA5‐115178, Invitrogen)
	Mouse anti‐HIF1 alpha	(MA1‐166504, Invitrogen)
Osteogenesis	Mouse anti‐ Collagen I	(NB600‐450, Novus Biologicals)
	Rabbit anti‐Osteocalcin	(PA5‐78871, Invitrogen)
	Rabbit anti‐PDGFRB	(MA5‐15143, Invitrogen)
	Rabbit anti‐Noggin	(BS‐2975R,Bioss)
	Rabbit anti‐SLIT3	(PA5‐104142, Invitrogen)

Secondary antibody		Catalogue number/company
Alexa Fluor™ 568 Goat anti‐Mouse		(A‐21144, Invitrogen)
Alexa Fluor™ 488 Donkey anti‐Rabbit		(A‐21206, Invitrogen)
Antibody, Alexa Fluor™ 568 Donkey anti‐Goat		(A‐11057, Invitrogen)

### 2.4 Statistical Analyses

Statistical analyses for comparing the results of the micro‐CT data made use of the One‐way analysis of variance (ANOVA) test followed by the Tukey test for multiple comparisons among groups. A significance threshold of *p* ≤ 0.05 was applied to determine statistical significance in all analyses. GraphPad Prism version 8.4.3 for Windows (GraphPad Software, San Diego, CA, USA) was used for both plotting and analyzing data points. Co‐localization data was analyzed through Pearson's correlation index (r) within the JACoP plugin, gauging the colocalization of selected marker pairs.

## 3 Results

### 3.1 Micro‐CT

**Figure 1:**
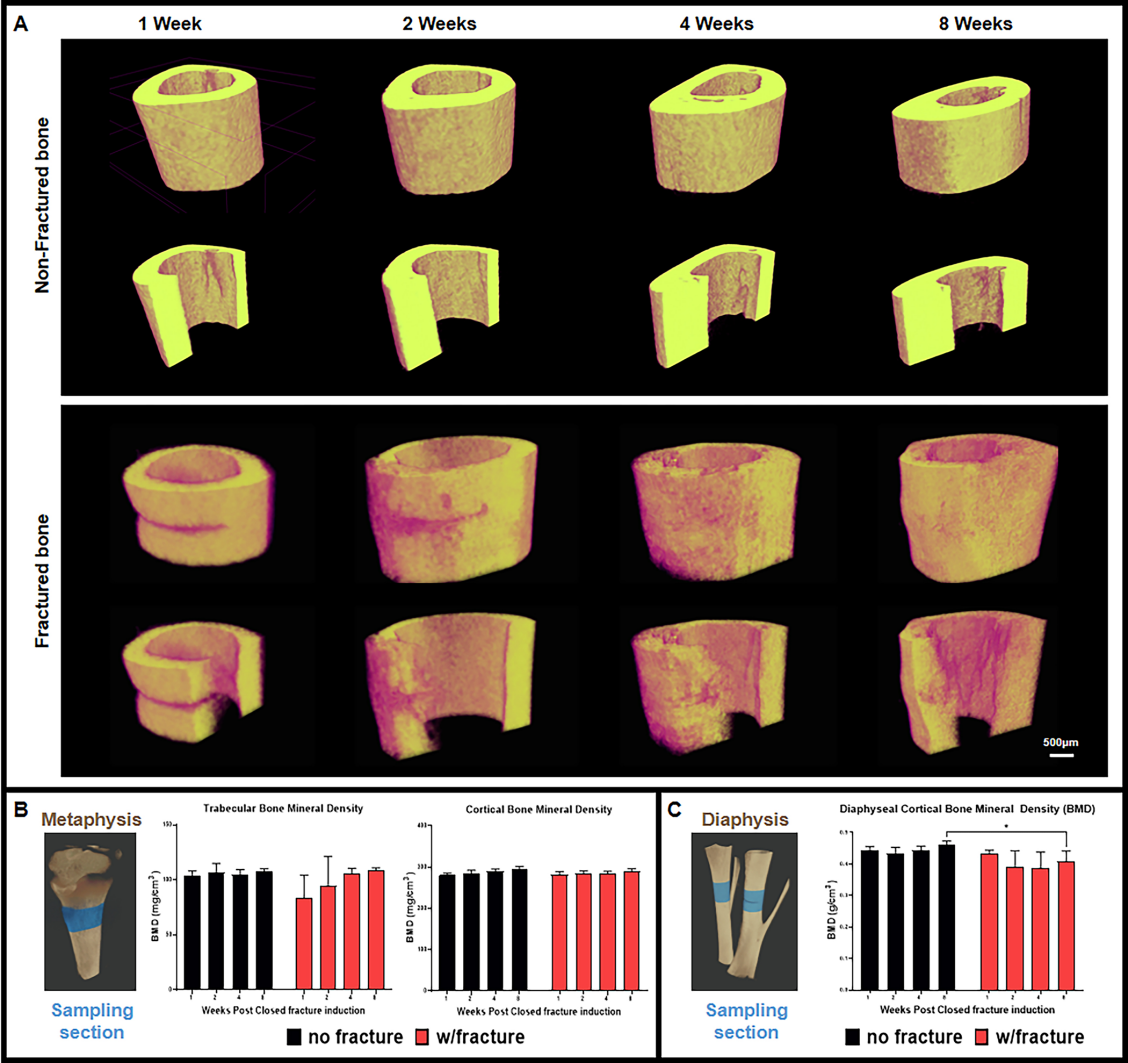
Whole and sectioned 3D reconstruction of Micro‐CT data of intact tibia bone tissue and fracture site sampled at 1 week, 2 weeks, 4 weeks, and 8 weeks (A). Bone mineral density (BMD) measurements comparing trabecular and cortical bone density along the metaphyseal area and (B) comparison of cortical BMD at the diaphyseal segment of the non‐fractured and fractured tibia bone tissue sampled at 1, 2, 4, and 8 weeks post‐fracture induction (Scale bar = 500 µm).

After sacrificing the animals at each observation time point (1, 2, 4, and 8 weeks post incomplete transverse fracture induction) micro‐CT scans of the tissue samples were taken to compare the integrity and bone mineral density of pristine bone and simulated an incomplete fracture. Malformed bone unions were no longer included in further analyses as this condition does not allow for proper identification of the simulated fracture line. At most, each group had 2 animals that were categorized for exclusion for further analyses. Figure 1A. shows the three‐dimensional section reconstructed from the Micro‐CT scans of the tissue samples taken at 1 week, 2 weeks, 4 weeks, and 8 weeks post‐fracture induction. Measurements of the trabecular and cortical bone densities taken along the proximal tibial metaphysis indicate no significant change in bone mineral densities between fractured (right) and non‐fractured bones (Figure 1B). However, measurements of cortical bone density along the fracture site on the right leg and the similar area on the left leg showed significantly lower bone mineral density at 8 weeks postfracture induction.

### 3.2 Regenerated Bone Tissue Morphology

**Figure 2:**
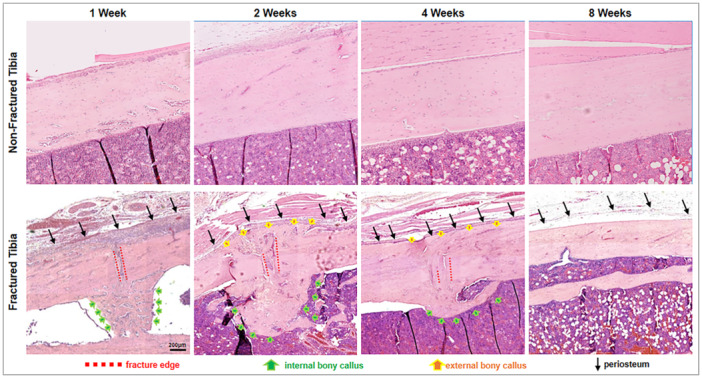
H&E stained tissue sections of the tibia bone fracture site taken at 1 week, 2 weeks, 4 weeks, and 8 weeks post‐fracture induction showing the tissue formation over time. Compared to the fractured bone, pristine bone tissue showed relatively low numbers of cells within the bone matrix and appeared relatively uneventful in terms of microstructural changes. Spongy bony callus can be observed within the medullary space after 1 week. Thickened and externally expanded bony callus can be observed at 2 weeks. Highly consolidated bony callus is observed at 4 weeks although the fracture gap is still highly discernable. No visible trace of the fracture can be observed at 8 weeks post‐fracture induction (Scale bar = 200 µm).

Morphological changes occurring to the tissues at the fracture site were observed using tissue sections stained with hematoxylin and eosin (H&E). Figure 2 shows the temporal progression of bone tissue formation within the simulated fracture site in the tibia bone samples. At 1 week postfracture induction, the fracture site was readily identifiable due to the interruption of the lamellar lines that characterize normal bone tissue. A substantial influx of various cell types was observed within the fracture gap. This is accompanied by the formation of extensive spongy internal bony callus within the medullary cavity. The periosteal layer exhibited significant thickening, cell infiltration, and permeation of blood vessels. By 2 weeks postfracture, the spongy bony callus had also extended on the external surface of the cortical bone fracture site and begun to bridge and cover the existing gap. Although the fracture site remained visible, the periosteal layer had markedly thinned compared to the 1‐week time point. The internal callus within the medullary space continued to thicken, indicative of ongoing bone formation. Four weeks postfracture, both external and internal bony callus had undergone significant maturation, acquiring a morphology similar to that of native bone tissue. The lamellar lines of the newly formed bone aligned with those of the surrounding intact bone, although the fracture gap remained noticeable due to the opposing directions of the lines. The external new bone formation converged with the pattern of the native cortical bone, while the new bone within the fracture gap and medullary space resembled highly thickened and compact trabecular bone. By 8 weeks postfracture, the fracture site was no longer discernible. The formation initially observed at 4 weeks had fully integrated with the surrounding cortical bone tissue, leaving no visible trace of the initial fracture. These findings demonstrate the dynamic process of bone fracture healing, characterized by a cascade of cellular and molecular events that ultimately lead to the restoration of bone integrity.

### 3.3 Angiogenesis in Incomplete Transverse Bone Fracture

**Figure 3:**
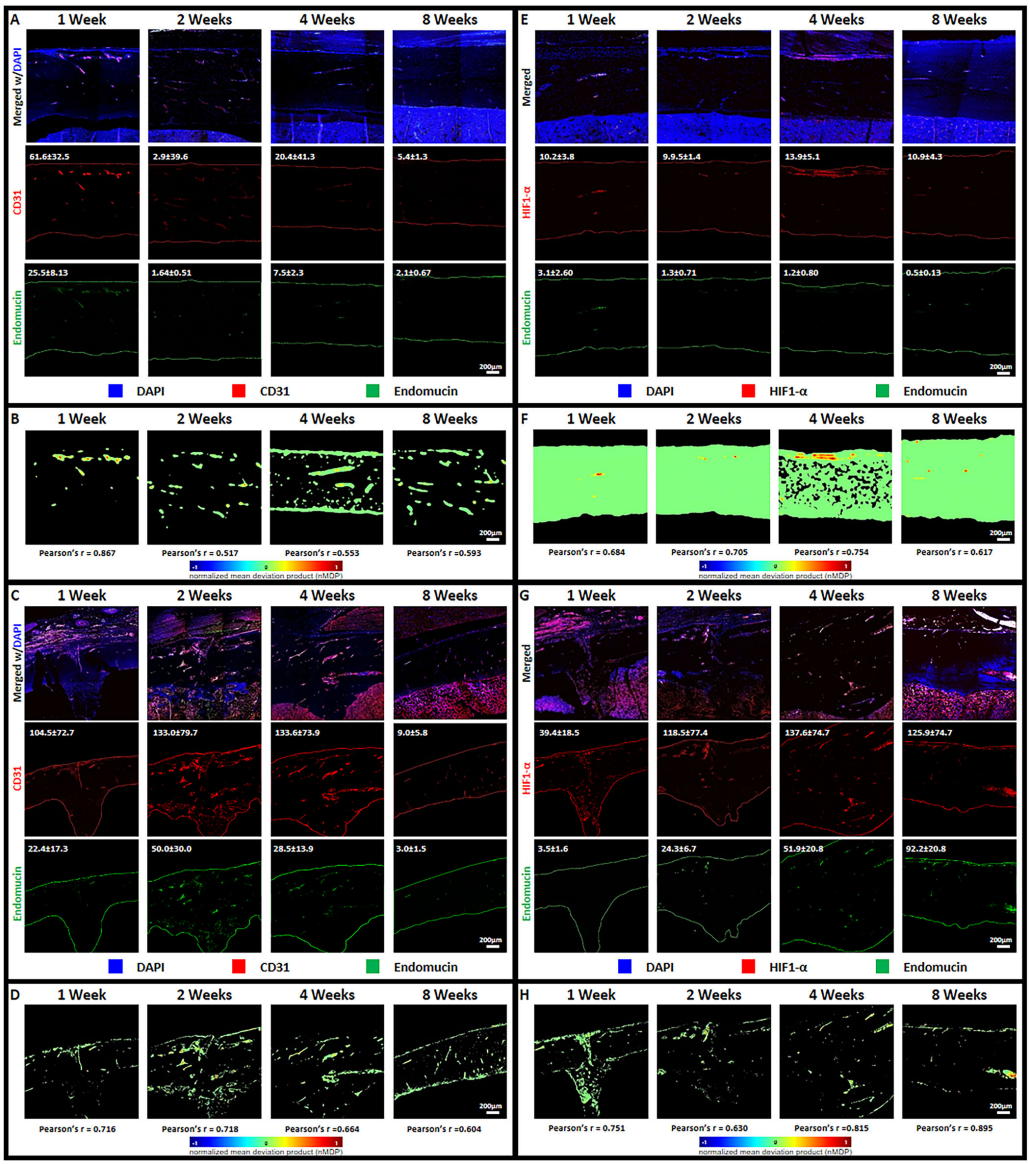
Confocal images of sectioned tissue samples of tibia fracture site stained for observing angiogenesis: CD31 paired with Endomucin for identification of H type blood vessels (A‐control group; C‐fracture group) and HIF1‐α paired with Endomucin (E‐control group; G‐fracture group) (Fluorescence intensity value = inset number). Confocal images of Colocalization color map of CD31 paired with Endomucin (B‐control group; d‐fracture group) and HIF1‐α and endomucin (F‐control group; H‐fracture group) with corresponding calculated Pearson's r value (Scale bar = 200 µm).

To investigate the dynamics of angiogenesis within fracture sites, we focused on H type blood vessels and the expression of Hypoxia‐inducible factor 1‐alpha (HIF1‐α). The persistence of H type blood vessels over time was determined by observing tissue sections stained for CD31 and endomucin. Figure 3A presents confocal images of sectioned tibia fracture sites at 1, 2, 4, and 8 weeks postfracture induction. These images revealed the decreasing presence of H type blood vessels throughout the observation time points. Fluorescence intensity measurements of CD31, an endothelial cell surface marker, demonstrated consistent expression throughout the observation period, suggesting a stable presence of blood vessels. In contrast, endomucin, another endothelial marker, exhibited a peak in expression around 2 weeks postfracture induction, followed by a gradual decline. This pattern indicates that while H type blood vessels are initially abundant, their density may decrease over time. Pearson's correlation analysis between CD31 and endomucin revealed a decreasing correlation over the 8‐week observation period (Figure 3B). This finding further supports the notion that while H type blood vessels are present throughout the healing process, their composition from the overall vasculature may change. To explore the relationship between angiogenesis and HIF1‐α expression, we conducted paired observations of HIF1‐α and endomucin, taking into consideration previous studies noting increased expression of endomucin for identifying the presence of H type blood vessels in bone tissue [39, 40, 41, 42] and HIF1‐α as a potent regulator of angiogenesis [25, 26, 43, 44, 45]. Figure 3C shows confocal images of these paired observations. Fluorescence intensity measurements revealed that HIF1‐α expression gradually peaked at 4 weeks postfracture induction and then stabilized at a level similar to that observed at 2 weeks. Correlation analyses between HIF1‐α and endomucin demonstrated an increasing Pearson's correlation over the 8‐week observation period. This suggests a decreasing association between HIF1‐α and H type blood vessels during the healing process. The increased colocalization of HIF1‐α and endomucin may indicate that HIF1‐α plays a role in the maintenance or remodeling of H type blood vessels in the early stages of fracture healing.

### 3.4 Osteogenesis in Non‐Stabilized Incomplete Transverse Bone Fracture

**Figure 4:**
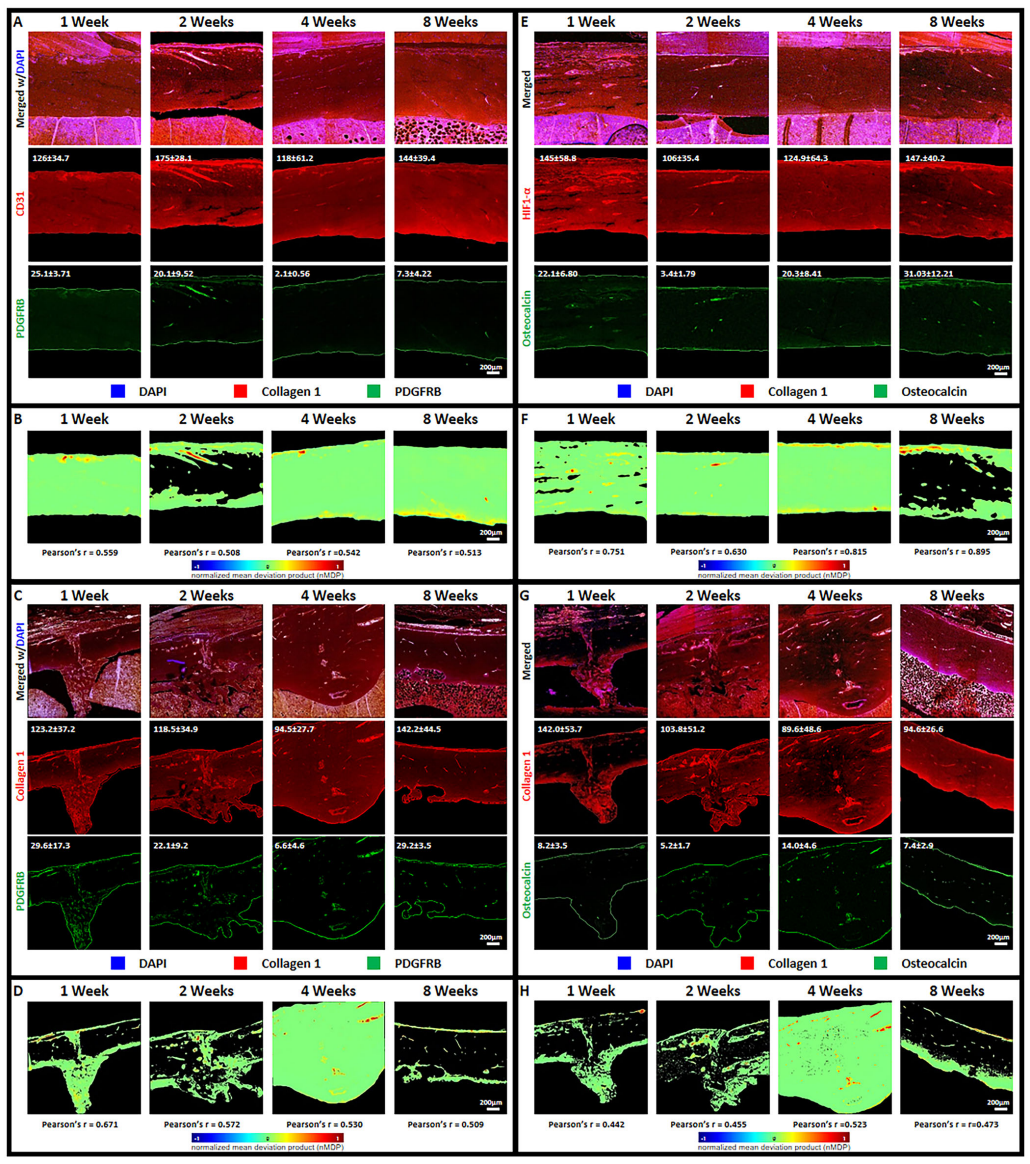
Confocal images of sectioned tissue samples of tibia fracture site stained for observing initial and late osteogenesis: collagen 1 paired with PDGFRB (A‐control group; C‐fracture group) and collagen 1 paired with Osteocalcin (E‐control group; G‐fracture group) (Fluorescence intensity value = inset number). Confocal images of Colocalization color map of collagen 1 paired with PDGFRB (B‐control group; d‐fracture group) and collagen 1 and osteocalcin (F‐control group; H‐fracture group) with corresponding calculated Pearson's r value (Scale bar = 200 µm).

To investigate osteogenesis within fracture sites, tissue sections were stained for collagen 1, PDGFRB, and osteocalcin. These proteins are involved in various stages of bone development and remodeling. Figure 4A presents confocal images of sectioned tibia fracture sites at 1, 2, 4, and 8 weeks postfracture induction, stained for PDGFRB expression. Fluorescence intensity measurements of collagen 1 demonstrated extensive expression in bone tissue throughout the observation period. In contrast, PDGFRB expression exhibited a decline at 4 weeks postfracture induction. Pearson's correlation analysis between collagen 1 and PDGFRB revealed a gradual decrease over the 8‐week observation period (Figure 4B). A decrease in PDGFRB, despite the continued presence of collagen 1 during healing, suggests a potential shift in cellular activity or a reduction in mesenchymal stem cell (MSC) involvement. PDGFRB activates intracellular signaling pathways (like PI3K and MAPK) that regulate cell proliferation, survival, migration, and differentiation. A decrease could lead to altered signaling and downstream cellular responses in various cell types. Changes in PDGFRB could reflect a shift in the overall growth factor signaling landscape, potentially favoring other receptor tyrosine kinases and their associated cellular activities. This is because PDGFRB is crucial for MSC identity and function in tissue repair and blood vessel support, but its presence also extends to other cells involved in vascular health and tissue remodeling [46, 47] The relationship between collagen 1 and osteocalcin in later stages of bone healing was also observed. Figure 4C shows confocal images of tissue sections stained for collagen 1 and osteocalcin. Fluorescence intensity measurements revealed a gradual increase in osteocalcin expression over time, while collagen 1 expression remained relatively constant. Pearson's correlation analysis between collagen 1 and osteocalcin showed a weak initial correlation (1–2 weeks postfracture induction), followed by a peak at 4 weeks and a subsequent decline to near‐initial levels by 8 weeks. This pattern suggests that the most intense tissue remodeling may occur around 4 weeks postfracture induction when there is a heightened association between collagen synthesis and osteoblast activity. These results are similar to that of previous findings from other studies where osteocalcin levels out upon resolving the fracture [48, 49].

### 3.5 Correlation of Developmental and Guidance Markers in Relation to Angiogenesis and Osteogenesis Non‐Stabilized Incomplete Transverse Bone Fracture

**Figure 5:**
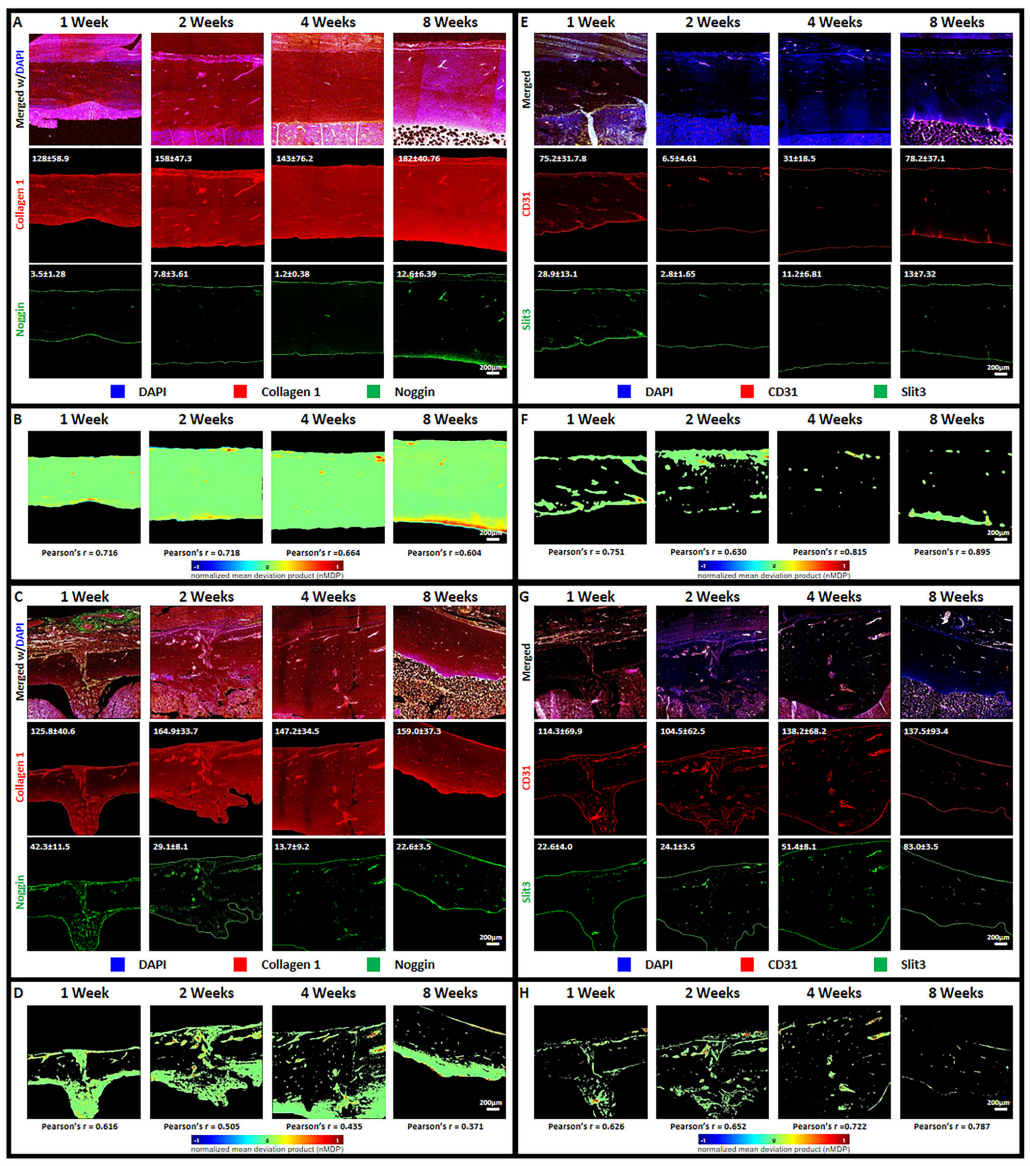
Confocal images of sectioned tissue samples of tibia fracture site stained for observing developmental and guidance markers of bone tissue formation: collagen 1 paired with noggin (A‐control group; C‐fracture group) and CD31 paired with Slit3 (E‐control group; G‐fracture group) (Fluorescence intensity value = inset number). Confocal images of Colocalization color map of collagen 1 paired with noggin (B‐control group; d‐fracture group) and CD31 and Slit3 (F‐control group; H‐fracture group) with corresponding calculated Pearson's r value (Scale bar = 200 µm).

To further explore the regulatory mechanisms underlying angiogenesis and osteogenesis in bone fracture healing, we examined the expression of markers related to tissue development and guidance. Noggin and SLIT3 markers were chosen for this analysis. Figure 5 presents confocal images of sectioned tibia fracture sites stained for collagen 1 paired with Noggin (Figure 5A) and CD31 paired with SLIT3 (Figure 5B) at 1, 2, 4, and 8 weeks postfracture induction. As previously observed in our analysis of angiogenesis and osteogenesis, the expression of collagen 1 (Figure 5A) and CD31 (Figure 5B) remained relatively consistent throughout the healing process. Fluorescence intensity measurements revealed that Noggin expression peaked at 1‐week post‐fracture induction and subsequently declined to a minimum at 4 weeks, recovering slightly by 8 weeks. In contrast, SLIT3 expression was initially low but exhibited a noticeable increase from 2 to 4 weeks, maintaining elevated levels until 8 weeks. Pearson's correlation analysis between collagen 1 and Noggin showed a consistently weak correlation across all observation points. This suggests a limited association between these two markers throughout the healing process. On the other hand, the correlation between CD31 and SLIT3 exhibited a slight elevation at 4 weeks, which persisted until 8 weeks. This finding indicates a potential interaction between retained vasculature and SLIT3 signaling during later stages of fracture healing.

### 3.6 Temporal Expression of Angiogenic and Osteogenic in Long Bone Fracture Site

**Figure 6:**
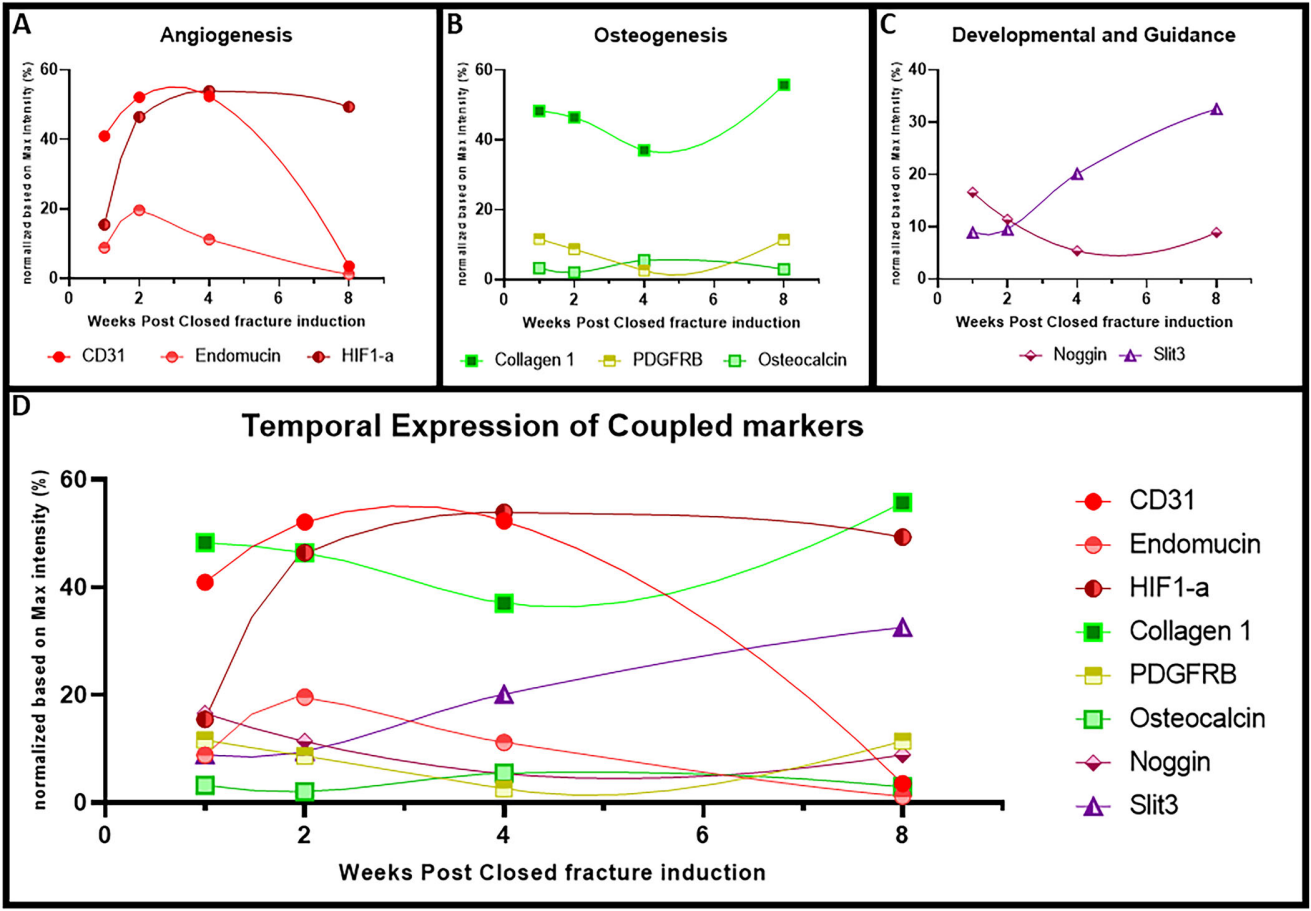
Expression levels based on fluorescence intensity measurements (normalized relative to maximum gray value) of angiogenic (A), osteogenic (B), and developmental and guidance (C) markers were plotted using tissue samples from an incomplete transverse fracture animal model. The integrated temporal plot (D) highlights key time points where significant shifts in marker expression levels occur across the aforementioned group.

Fluorescence intensity data from confocal images were normalized against the maximum grayscale value and were used as a reference for establishing the temporal expression of the different markers. Figure 6 illustrates the temporal expression patterns of angiogenic (CD31, endomucin, HIF1‐α), osteogenic (collagen 1, PDGFRB, osteocalcin), developmental (Noggin), and guidance markers (SLIT3) at the fracture site over 8 weeks. Angiogenic markers such as CD31 and endomucin exhibited a gradual increase followed by a decline, suggesting initial vascularization and subsequent remodeling. HIF1‐α expression remained elevated after consolidation, which may indicate that HIF1‐α plays a different role in bone tissue other than the formation of blood vessels during the early stage of fracture healing. Osteogenic markers PDGFRB and osteocalcin showed inverse levels of expression at 4 weeks, suggesting a dynamic interplay between the early and late stages of bone regeneration. Collagen 1 expression followed a similar trend, indicating initial matrix deposition with subsequent remodeling phase. Developmental and guidance markers Noggin and SLIT3 experienced transient decrease and increase respectively, suggesting their opposing roles in early‐stage fracture healing or regulation of cellular migration.

## 4 Discussion

Bone regeneration is a crucial process by which the main supporting structure of the human body is maintained. Unlike the complex structures of the skull, where fractures also demand regeneration [10, 50, 51, 52, 53], the robust nature and weight‐bearing function of long bones underscore the profound importance of effective bone regeneration for maintaining mobility and overall physical well‐being. This study investigates the temporal dynamics of fracture healing, specifically examining the persistence of H type blood vessels, the expression patterns of angiogenic and osteogenic markers over time, and their correlation with distinct stages of bone repair in a non‐scaffold supplemented fracture model. The current study did not include other modes of observation such as protein assay and flow cytometry but instead focused on image‐based analyses to accommodate initial testing such as micro‐CT scanning and gross tissue morphology. Both aforementioned methods were no longer considered to conserve the number of animals used and maximize the samples for downstream image analyses. Initial characterization analyzing the metaphyseal segment of the control and fracture leg using micro‐CT scans revealed that overall bone structure and mineral content remained largely unchanged in both groups, suggesting the fracture did not significantly impact overall bone quality. However, localized micro‐CT analysis along the diaphyseal segment demonstrated a significant decrease in cortical bone density in the operated leg compared to the similar region in the non‐operated leg at 8 weeks. This finding aligns with previous research [54, 55], indicating that fracture‐induced disruption of bone remodeling and the presence of less mineralized callus formation can lead to localized bone density reductions. Over time, as the callus matures and undergoes remodeling, its mineral density may increase to approach that of the surrounding intact bone [56, 57, 58].

H&E‐stained tissue sections further supported these observations. Changes in lamellar structures were evident within the evolving callus, demonstrating distinct morphological features at different time points. Notably, at 8 weeks postfracture, the previously observed callus had fully consolidated with the original bone tissue, indicating successful bone repair.

Type H blood vessels, distinguished by their high expression of endomucin and CD31, are typically found in the growth plate and metaphysis, where they play a crucial role in both bone development and repair [4, 5, 6, 7, 59, 60] and are known to develop in bone fractures. While their importance in the early stages of callus formation is well‐documented, their behavior in the later phases of fracture healing is less understood. Some research indicates their continued involvement in bone remodeling and the maintenance of a regenerative microenvironment. Another key marker analyzed in this context was HIF‐1α, a transcription factor activated by low oxygen levels. HIF‐1α plays a critical role in fracture healing by stimulating angiogenesis and enhancing osteogenesis coupling vascularization with bone formation [7, 25, 61, 62, 63, 64]. HIF‐1α induces the expression of Vascular Endothelial Growth Factor (VEGF), a potent angiogenic factor that stimulates endothelial cell proliferation and migration, leading to the formation of new blood vessels essential for nutrient and oxygen supply to the healing tissue [45, 63]. Simultaneously, HIF‐1α can also enhance osteogenesis by promoting the differentiation and activity of osteoblasts through the upregulation of factors like BMPs and Runx2 [62, 64]. This coordinated regulation ensures that the developing bone tissue is adequately vascularized, a crucial aspect of successful bone regeneration.

Ideally, the levels of HIF‐1α are lowered as oxygenation levels are restored [62, 65, 66]. This is in contrast with our current results wherein the HIF‐1α level maintained a relatively high even at 8 weeks post‐fracture, which might indicate the need for supportive factors even after full consolidation of the fracture site. Additionally, sustained HIF1‐α expression in regenerated bone may contribute to bone remodeling, which cycles between osteogenesis and osteolysis [67, 68] through regulation of RANKL/OPG ratios that affects osteoclast activity [69, 70].

Markers related to bone formation were observed to establish osteogenesis in the sampled tissues. Previous studies have shown that fractures can alter the expression of collagen type I (Collagen1) in bone tissue [71, 72, 73]. During repair, osteoblasts produce collagen type I to rebuild the bone matrix, a process influenced by mechanical stress and microenvironmental changes [73]. Collagen type I expression initially increases but fluctuates during remodeling and consolidation, ultimately decreasing as the bone matures [71, 72, 73]. This dynamic regulation was evident from H&E staining and fluctuating fluorescence measurements of collagen 1 expression [71, 72].

PDGFRB serves as an early marker for bone formation at fracture sites, where its expression is typically elevated [22, 74, 75, 76]. It plays a key role in bone healing by promoting fibroblast and vascular mural cell proliferation and chemotaxis, facilitating extracellular matrix synthesis critical for tissue scaffolding [77, 78, 79, 80]. This increased PDGFRB expression recruits and differentiates skeletal stem/progenitor cells, aiding homeostasis and blood vessel formation essential for bone regeneration [77]. Additionally, PDGFRB influences bone remodeling by regulating osteoblast and osteoclast activity, impacting bone resorption and formation [78, 81, 82, 83]. It is expressed by osteoblast‐lineage cells and upregulates factors like colony‐stimulating factors, supporting bone resorption, and mesenchymal stem cell differentiation [21].

Osteocalcin, a bone‐forming protein produced by osteoblasts, is a key marker of bone mineralization [23, 84, 85]. Its expression varies across bone regions and during fracture healing. While elevated in the early stages, osteocalcin levels may not consistently increase with fracture consolidation, suggesting a dynamic role in bone remodeling [24]. Our observations, including collagen 1 staining, support this, indicating peak remodeling around 4 weeks postfracture. This understanding contributes to our knowledge of bone fracture repair and may guide future therapeutic strategies.

During bone regeneration, collagen type I forms the essential structural scaffold for new bone tissue and supports cell attachment [86, 87]. PDGFRB signaling orchestrates the recruitment and proliferation of mesenchymal stem cells and promotes angiogenesis, ensuring adequate blood supply [88]. As osteoblasts mature within this collagenous matrix, they secrete osteocalcin, a protein that contributes to matrix organization and potentially regulates mineralization [89]. The coordinated interplay between collagen I providing the framework, PDGFRB guiding cellular events and vascularization, and osteocalcin influencing matrix quality and mineralization is crucial for successful bone repair and the formation of functional bone.

To further investigate the interplay between angiogenesis and osteogenesis, markers for tissue development and guidance (Noggin and SLIT3) were analyzed within the fracture site. Noggin, known to be released from H type blood vessels [31, 32, 33], and SLIT3, secreted by osteoblasts and osteoclasts [31, 32, 33], were chosen for their potential to provide insights into both processes. This pairing is a form of cross‐referencing for the synergistic effect of angiogenesis and osteogenesis., Fluorescence intensity measurements revealed an initial increase in Noggin expression in fractures, consistent with previous findings demonstrating its role in regulating BMP activity during early callus formation [31, 32, 33, 90, 91]. Noggin acts as a BMP antagonist, inhibiting bone formation and resorption [91]. While crucial for early callus formation, excessive Noggin can hinder healing [91, 92, 93, 94]. Conversely, SLIT3 expression was initially low at the fracture site but increased as osteoclast activity and bone healing progressed [31, 32, 33]. SLIT3, derived from osteoblasts or osteoclasts, promotes bone formation by activating signaling pathways that enhance H type vessel formation and stimulate cell migration [4, 5, 39, 95, 96]. These findings suggest a complex interplay between Noggin and SLIT3 in regulating angiogenesis and osteogenesis during fracture healing.

The relationship between Noggin and SLIT3 in bone fracture healing is complex. While Noggin expression peaks transiently, Slit3 expression increases steadily, correlating with angiogenesis (CD31). This suggests opposing roles for these factors in bone remodeling. Noggin's limited correlation with collagen 1 indicates a less direct influence on osteogenesis. In contrast, Slit3's sustained increase suggests involvement in angiogenesis and potentially other processes. Key changes in marker expression occur at 2 and 4 weeks postfracture. Some markers (HIF1‐α, SLIT3, Osteocalcin) remain elevated even after consolidation, suggesting long‐term effects of the injury. Our study highlights the importance of H type blood vessels throughout healing, with HIF1‐α playing a crucial role in their regulation. Collagen 1 expression persists, reflecting ongoing matrix deposition. PDGFRB and osteocalcin expression dynamics suggest a dynamic interplay between cell proliferation, differentiation, and mineralization. The peak in collagen 1 and osteocalcin expression around 4 weeks indicates maximal bone formation.

The study has several limitations. The rat model may not fully reflect human bone healing and vascularization, and the 8‐week observation period may miss long‐term remodeling. The focus on specific angiogenic and osteogenic markers may overlook other key pathways, and controlled lab conditions may not reflect real‐world healing factors. The persistence of HIF1‐α expression raises questions about its role, and the study does not fully explore immune cell involvement or cellular mechanisms, suggesting areas for future research. Although these results might relate to the role of HIF1‐α in the regulation of the RANKL/OPG ratio that influences osteoclast activity [69, 70], further experimentation would be required to confirm this theory. Lastly, the current study does not cover observations of similar phenomena in stratified age groups, limiting the current observations within the context of a young adult animal model.

## 5 Conclusions

This study advances our knowledge of how angiogenesis, bone growth, and regulatory molecules interact during bone fracture healing. These findings may inform the development of new treatments to improve bone repair and reduce fracture complications. Potential therapeutic targets include HIF1‐α and other factors influencing H type blood vessel formation, as well as Noggin and Slit3, which regulate angiogenesis and bone growth timing. Future studies should delve deeper into H type blood vessel regulation and their role in bone repair, including their interactions with other cell types (like immune cells and stem cells). Finally, investigating the impact of various therapies on H type blood vessel formation and bone repair is crucial for identifying effective clinical strategies.

## Author Contributions

Kyung Wook Kim, Andrew Reyes Padalhin, Celine Abueva, Phil‐Sang Chung, and Seung Hoon Woo conceptualized the study. Kyung Wook Kim, Andrew Reyes Padalhin, Hyun Seok Ryu, Seung Hyeon Yoo, Hwee Hyon Seo, and So Young Park developed the methodology. Andrew Reyes Padalhin, Hyun Seok Ryu, and Seung Hyeon Yoo performed the formal analysis. The investigation was carried out by Kyung Wook Kim, Andrew Reyes Padalhin, Seung Hyeon Yoo, Jun Sang Bae, Hwee Hyon Seo, So Young Park, and Hyun Sik Gong Resources were provided by Kyung Wook Kim, Seung Hyeon Yoo, Celine Abueva, Jun Sang Bae, Hwee Hyon Seo, Phil‐Sang Chung, and Seung Hoon Woo Andrew Reyes Padalhin, Hyun Seok Ryu, Phil‐Sang Chung, and Seung Hoon Woo prepared the original draft of the manuscript. The writing – review and editing was done by Kyung Wook Kim, Andrew Reyes Padalhin, Phil‐Sang Chung, Hyun Sik Gong, and Seung Hoon Woo Visualization efforts were conducted by Andrew Reyes Padalhin, So Young Park, and Seung Hyeon Yoo Supervision was managed by Celine Abueva, Jun Sang Bae, Phil‐Sang Chung, Hyun Sik Gong, and Seung Hoon Woo The project was administered by Phil‐Sang Chung, Hyun Sik Gong, and Seung Hoon Woo Funding for the project was acquired by Phil‐Sang Chung, Hyun Sik Gong, and Seung Hoon Woo All authors have read and agreed to the published version of the manuscript.

## Conflicts of Interest

The authors declare no conflicts of interest.

## Data Availability

Data available on request from the authors.

## References

[1] J. A. Buckwalter , M. J. Glimcher , R. R. Cooper , and R. Recker , “Bone Biology. I: Structure, Blood Supply, Cells, Matrix, and Mineralization,” Instructional Course Lectures 45 (1996): 371–386. 8727757

[2] B. Clarke , “Normal Bone Anatomy and Physiology,” supplement, Clinical Journal of the American Society of Nephrology 3, no. S3 (2008): S131–S139. 18988698 10.2215/CJN.04151206 PMC3152283

[3] R. Florencio‐Silva , G. R. S. Sasso , E. Sasso‐Cerri , M. J. Simões , and P. S. Cerri , “Biology of Bone Tissue: Structure, Function, and Factors That Influence Bone Cells,” BioMed Research International 2015 (2015): 1–17. 10.1155/2015/421746 PMC4515490 26247020

[4] J. Zhang , J. Pan , and W. Jing , “Motivating Role of Type H Vessels in Bone Regeneration,” Cell Proliferation 53, no. 9 (2020): e12874. 33448495 10.1111/cpr.12874 PMC7507571

[5] J. Xu , S. He , T. Xia , Y. Shan , and L. Wang , “Targeting Type H Vessels in Bone‐Related Diseases,” Journal of Cellular and Molecular Medicine 28, no. 4 (2024): e18123. 38353470 10.1111/jcmm.18123 PMC10865918

[6] Z. Ruan , H. Yin , T. F. Wan , et al., “Metformin Accelerates Bone Fracture Healing by Promoting Type H Vessel Formation Through Inhibition of YAP1/TAZ Expression,” Bone Research 11, no. 1 (2023): 45. 37587136 10.1038/s41413-023-00279-4 PMC10432554

[7] A. P. Kusumbe , S. K. Ramasamy , and R. H. Adams , “Coupling of Angiogenesis and Osteogenesis by a Specific Vessel Subtype in Bone,” Nature 507, no. 7492 (2014): 323–328. 24646994 10.1038/nature13145 PMC4943525

[8] R. Vasileva and T. Chaprazov , “Bone Healing of Critical‐Sized Femoral Defects in Rats Treated With Erythropoietin Alone or in Combination With Xenograft,” Veterinary Sciences 10, no. 3 (2023): 196. 36977235 10.3390/vetsci10030196 PMC10056540

[9] J. Bai , L. Li , N. Kou , et al., “Low Level Laser Therapy Promotes Bone Regeneration by Coupling Angiogenesis and Osteogenesis,” Stem Cell Research & Therapy 12, no. 1 (2021): 432. 34344474 10.1186/s13287-021-02493-5 PMC8330075

[10] M. K. Baek , J. H. Jung , S. T. Kim , and I. G. Kang , “Delayed Treatment of Zygomatic Tetrapod Fracture,” Clinical and Experimental Otorhinolaryngology 3, no. 2 (2010): 107–109. 20607081 10.3342/ceo.2010.3.2.107 PMC2896732

[11] A. Kammerer , F. A. Hartmann , C. Nau , et al., “The Impact of Defect Size on Bone Healing in Critical‐Size Bone Defects Investigated on a Rat Femur Defect Model Comparing two Treatment Methods,” Bioengineering 11, no. 3 (2024): 287. 38534561 10.3390/bioengineering11030287 PMC10968167

[12] Y. Chen , J. Wu , F. Li , L. Ye , and H. Wang , “Creating a box‐Cavity Defect Model in the Cortical Bone of Rat Femora,” Journal of Visualized Experiments 21, no. 201 (2023): e66068. 10.3791/66068 38078597

[13] P. P. Spicer , J. D. Kretlow , S. Young , J. A. Jansen , F. K. Kasper , and A. G. Mikos , “Evaluation of Bone Regeneration Using the rat Critical Size Calvarial Defect,” Nature Protocols 7, no. 10 (2012): 1918–1929. 23018195 10.1038/nprot.2012.113 PMC3513397

[14] Y. Sun , H. Helmholz , and R. Willumeit‐Römer , “Surgical Classification for Preclinical rat Femoral Bone Defect Model: Standardization Based on Systematic Review, Anatomical Analysis and Virtual Surgery,” Bioengineering 9, no. 9 (2022): 476. 36135022 10.3390/bioengineering9090476 PMC9495991

[15] Z. Zhang , Y. Gan , Y. Guo , X. Lu , and X. Li , “Animal Models of Vertical Bone Augmentation (Review),” Experimental and Therapeutic Medicine 22, no. 3 (2021): 919. 34335880 10.3892/etm.2021.10351 PMC8290405

[16] J.‐H. Kim and H.‐W. Kim , “Rat Defect Models for Bone Grafts and Tissue Engineered Bone Constructs,” Tissue Engineering and Regenerative Medicine 10, no. 6 (2013): 310–316.

[17] R. Kambe , K. Mitomo , T. Ikarashi , et al., “Localization of Both CD31‐ and Endomucin‐Expressing Vessels in Mouse Dental Pulp,” Acta Histochemica et Cytochemica 57, no. 5 (2024): 157–163. 39552934 10.1267/ahc.24-00009 PMC11565222

[18] J. Wang , Y. Gao , P. Cheng , et al., “CD31hiEmcnhi Vessels Support New Trabecular Bone Formation at the Frontier Growth Area in the Bone Defect Repair Process,” Scientific Reports 7, no. 1 (2017): 4990. 28694480 10.1038/s41598-017-04150-5 PMC5504063

[19] X. Li , Q. Lv , P. Liu , G. Han , and S. Yu , “Understanding of Endomucin: A Multifaceted Glycoprotein Functionality in Vascular Inflammatory‐Related Diseases, Bone Diseases and Cancers,” Advanced Biology 8, no. 10 (2024): 2400061. 10.1002/adbi.202400061 38955667

[20] V. Crenn , J. Amiaud , A. Gomez‐Brouchet , et al., “Signature of the Vascular Tumor Microenvironment as a Marker of the Therapeutic Response to Doxorubicin in a Preclinical Model of Osteosarcoma,” American Journal of Cancer Research 12 (2022): 1843–1854. 35530297 PMC9077066

[21] J. Brun , C. M. Andreasen , C. Ejersted , T. L. Andersen , J. Caverzasio , and C. Thouverey , “PDGF Receptor Signaling in Osteoblast Lineage Cells Controls Bone Resorption Through Upregulation of Csf1 Expression,” Journal of Bone and Mineral Research 35, no. 12 (2020): 2458–2469. 32777109 10.1002/jbmr.4150

[22] F. Wang , Y. Ye , Z. Zhang , et al., “PDGFR in PDGF‐BB/PDGFR Signaling Pathway Does Orchestrates Osteogenesis in a Temporal Manner,” Research; a Journal of Science and its Applications 6 (2023): 0086. 10.34133/research.0086 PMC10202377 37223474

[23] M. L. Zoch , T. L. Clemens , and R. C. Riddle , “New Insights Into the Biology of Osteocalcin,” Bone 82 (2016): 42–49. 26055108 10.1016/j.bone.2015.05.046 PMC4670816

[24] R. S. Dradjat , P. Sananta , R. D. Rosandi , and L. D. Siahaan , “Osteocalcin Biomarker Level Evaluation on Fracture Healing With Bone Defect After Stromal Vascular Fraction Application in Murine Model,” Annals of Medicine and Surgery 71 (2021): 103020. 34840768 10.1016/j.amsu.2021.103020 PMC8606847

[25] W. Chen , P. Wu , F. Yu , G. Luo , L. Qing , and J. Tang , “HIF‐1α Regulates Bone Homeostasis and Angiogenesis, Participating in the Occurrence of Bone Metabolic Diseases,” Cells 11, no. 22 (2022): 3552. 36428981 10.3390/cells11223552 PMC9688488

[26] S. Song , G. Zhang , X. Chen , et al., “HIF‐1α Increases the Osteogenic Capacity of ADSCs by Coupling Angiogenesis and Osteogenesis via the HIF‐1α/VEGF/AKT/mTOR Signaling Pathway,” Journal of Nanobiotechnology 21, no. 1 (2023): 257. 37550736 10.1186/s12951-023-02020-z PMC10405507

[27] W. Zhang , F. Yang , Q. Yan , et al., “Hypoxia Inducible factor‐1α Related Mechanism and TCM Intervention in Process of Early Fracture Healing,” Chinese Herbal Medicines 16, no. 1 (2024): 56–69. 38375046 10.1016/j.chmed.2023.09.006 PMC10874770

[28] H. Peng , A. Usas , D. Hannallah , A. Olshanski , G. M. Cooper , and J. Huard , “Noggin Improves Bone Healing Elicited by Muscle Stem Cells Expressing Inducible BMP4,” Molecular Therapy 12, no. 2 (2005): 239–246. 16043095 10.1016/j.ymthe.2005.02.027

[29] D. C. Wan , J. H. Pomerantz , L. J. Brunet , et al., “Noggin Suppression Enhances In Vitro Osteogenesis and Accelerates In Vivo Bone Formation,” Journal of Biological Chemistry 282, no. 36 (2007): 26450–26459. 17609215 10.1074/jbc.M703282200

[30] K. Truchan and A. M. Osyczka , “Noggin Promotes Osteogenesis in Human Adipose‐Derived Mesenchymal Stem Cells via FGFR2/Src/Akt and ERK Signaling Pathway,” Scientific Reports 14, no. 1 (2024): 6724. 38509118 10.1038/s41598-024-56858-w PMC10954655

[31] B. J. Kim , Y. S. Lee , S. Y. Lee , et al., “Osteoclast‐Secreted SLIT3 Coordinates Bone Resorption and Formation,” Journal of Clinical Investigation 128, no. 4 (2018): 1429–1441. 29504949 10.1172/JCI91086 PMC5873876

[32] A. R. Yallowitz , J. H. Shim , R. Xu , and M. B. Greenblatt , “An Angiogenic Approach to Osteoanabolic Therapy Targeting the SHN3‐SLIT3 Pathway,” Bone 172 (2023): 116761. 37030497 10.1016/j.bone.2023.116761 PMC10198948

[33] H. Kim , Y. J. Choi , Y. S. Lee , et al., “SLIT3 Regulates Endochondral Ossification by β‐Catenin Suppression in Chondrocytes,” Biochemical and Biophysical Research Communications 506, no. 4 (2018): 847–853. 30389141 10.1016/j.bbrc.2018.10.167

[34] N. Cesarovic , F. Nicholls , A. Rettich , et al., “Isoflurane and Sevoflurane Provide Equally Effective Anaesthesia in Laboratory Mice,” Laboratory Animals 44, no. 4 (2010): 329–336. 20507878 10.1258/la.2010.009085

[35] S. E. Raper , M. E. Barker , S. J. Burwen , and A. L. Jones , “Isoflurane as an Anesthetic for Experimental Animal Surgery,” Anatomical Record 218, no. 2 (1987): 116–122. 3619078 10.1002/ar.1092180204

[36] N. Aravindan , J. P. Cata , L. Hoffman , et al., “Effects of Isoflurane, Pentobarbital, and Urethane on Apoptosis and Apoptotic Signal Transduction in Rat Kidney,” Acta Anaesthesiologica Scandinavica 50, no. 10 (2006): 1229–1237. 16978161 10.1111/j.1399-6576.2006.01102.x

[37] S. Bolte and F. P. CordeliÈRes , “A Guided Tour Into Subcellular Colocalization Analysis in Light Microscopy,” Journal of Microscopy 224, no. 3 (2006): 213–232. 17210054 10.1111/j.1365-2818.2006.01706.x

[38] A. Gorlewicz , K. Krawczyk , A. A. Szczepankiewicz , P. Trzaskoma , C. Mulle , and G. M. Wilczynski , “Colocalization Colormap–An Imagej Plugin for the Quantification and Visualization of Colocalized Signals,” Neuroinformatics 18, no. 4 (2020): 661–664. 32361813 10.1007/s12021-020-09465-9

[39] Y. Peng , S. Wu , Y. Li , and J. L. Crane , “Type H Blood Vessels in Bone Modeling and Remodeling,” Theranostics 10, no. 1 (2020): 426–436. 31903130 10.7150/thno.34126 PMC6929606

[40] L. Marger , N. Liaudet , S. S. Scherrer , et al., “Identification of Type‐H‐Like Blood Vessels in a Dynamic and Controlled Model of Osteogenesis in Rabbit Calvarium,” Materials 15, no. 13 (2022): 4703. 35806828 10.3390/ma15134703 PMC9267487

[41] L. Wang , F. Zhou , P. Zhang , et al., “Human Type H Vessels are a Sensitive Biomarker of Bone Mass,” Cell Death & Disease 8, no. 5 (2017): e2760. 28471445 10.1038/cddis.2017.36 PMC5520742

[42] M. Yang , C. J. Li , Y. Xiao , et al., “Ophiopogonin D Promotes Bone Regeneration by Stimulating CD31hiEMCNhi Vessel Formation,” Cell Proliferation 53, no. 3 (2020): e12784. 32080957 10.1111/cpr.12784 PMC7106967

[43] L. Bentovim , R. Amarilio , and E. Zelzer , “HIF1α Is a Central Regulator of Collagen Hydroxylation and Secretion Under Hypoxia During Bone Development,” Development 139, no. 23 (2012): 4473–4483. 23095889 10.1242/dev.083881

[44] J. Wlodarczyk , A. Leng , S. N. Abadchi , et al., “Transfection of Hypoxia‐Inducible Factor‐1α mRNA Upregulates the Expression of Genes Encoding Angiogenic Growth Factors,” Scientific Reports 14, no. 1 (2024): 6738. 38509125 10.1038/s41598-024-54941-w PMC10954730

[45] C. Wan , S. R. Gilbert , Y. Wang , et al., “Activation of the Hypoxia‐Inducible Factor‐1α Pathway Accelerates Bone Regeneration,” Proceedings of the National Academy of Sciences 105, no. 2 (2008): 686–691. 10.1073/pnas.0708474105 PMC2206597 18184809

[46] S. Wang , M. Mo , J. Wang , et al., “Platelet‐Derived Growth Factor Receptor Beta Identifies Mesenchymal Stem Cells With Enhanced Engraftment to Tissue Injury and Pro‐Angiogenic Property,” Cellular and Molecular Life Sciences 75, no. 3 (2018): 547–561. 28929173 10.1007/s00018-017-2641-7 PMC11105282

[47] A. Tokunaga , T. Oya , Y. Ishii , et al., “PDGF Receptor β Is a Potent Regulator of Mesenchymal Stromal Cell Function,” Journal of Bone and Mineral Research 23, no. 9 (2008): 1519–1528. 18410236 10.1359/jbmr.080409

[48] S. Bailey , A. A. Poundarik , G. E. Sroga , and D. Vashishth , “Structural Role of Osteocalcin and its Modification in Bone Fracture,” Applied Physics Reviews 10, no. 1 (2023): 011410. 36915902 10.1063/5.0102897 PMC9999293

[49] T. Ohishi , M. Takahashi , A. Yamanashi , D. Suzuki , and A. Nagano , “Sequential Changes of Bone Metabolism in Normal and Delayed Union of the Spine,” Clinical Orthopaedics & Related Research 466, no. 2 (2008): 402–410. 18196424 10.1007/s11999-007-0054-x PMC2505135

[50] J. Kim , H. J. Jung , and W. S. Shim , “Corrective Septorhinoplasty in Acute Nasal Bone Fractures,” Clinical and Experimental Otorhinolaryngology 11, no. 1 (2018): 46–51. 28602065 10.21053/ceo.2017.00346 PMC5831659

[51] S. Y. Kim , Y. J. Kim , Y. H. Kim , and M. H. Park , “Audiologic Patterns of Otic Capsule Preserving Temporal Bone Fracture: Effects of the Affected Subsites,” Clinical and Experimental Otorhinolaryngology 9, no. 3 (2016): 206–211. 27337953 10.21053/ceo.2015.01116 PMC4996100

[52] J.‐H. Shin , S. Park , S. H. Baek , and S. Kim , “Cochlear Implantation After Bilateral Transverse Temporal Bone Fractures,” Clinical and Experimental Otorhinolaryngology 1, no. 3 (2008): 171–173. 19434252 10.3342/ceo.2008.1.3.171 PMC2671745

[53] J. Y. Kim , G. Choi , and J. H. Kwon , “Transantral Orbital Floor Fracture Repair Using a Folded Silastic Tube,” Clinical and Experimental Otorhinolaryngology 8, no. 3 (2015): 250–255. 26330920 10.3342/ceo.2015.8.3.250 PMC4553356

[54] B. Osipov , A. J. Emami , and B. A. Christiansen , “Systemic Bone Loss After Fracture,” Clinical Reviews in Bone and Mineral Metabolism 16, no. 4 (2018): 116–130. 31363348 10.1007/s12018-018-9253-0 PMC6667184

[55] X.‐Q. Zheng , J. Huang , J. Lin , and C. L. Song , “Pathophysiological Mechanism of Acute Bone Loss After Fracture,” Journal of Advanced Research 49 (2023): 63–80. 36115662 10.1016/j.jare.2022.08.019 PMC10334135

[56] H. Xu , Z. Xue , H. Ding , H. Qin , and Z. An , “Callus Formation and Mineralization After Fracture With Different Fixation Techniques: Minimally Invasive Plate Osteosynthesis Versus Open Reduction Internal Fixation,” PLoS One 10, no. 10 (2015): e0140037. 26444295 10.1371/journal.pone.0140037 PMC4596811

[57] Y. Liu , I. Manjubala , H. Schell , et al., “Size and Habit of Mineral Particles in Bone and Mineralized Callus During Bone Healing in Sheep,” Journal of Bone and Mineral Research 25, no. 9 (2010): 2029–2038. 20225262 10.1002/jbmr.84

[58] M. A. Gentile , D. Y. Soung , C. Horrell , R. Samadfam , H. Drissi , and L. T. Duong , “Increased Fracture Callus Mineralization and Strength in Cathepsin K Knockout Mice,” Bone 66 (2014): 72–81. 24928497 10.1016/j.bone.2014.04.032

[59] A. Zahr , P. Alcaide , J. Yang , et al., “Endomucin Prevents Leukocyte–Endothelial Cell Adhesion and has a Critical Role Under Resting and Inflammatory Conditions,” Nature Communications 7, no. 1 (2016): 10363. 10.1038/ncomms10363 PMC4740757 26831939

[60] G. Zhang , X. Yang , and R. Gao , “Research Progress on the Structure and Function of Endomucin,” Animal Models and Experimental Medicine 3, no. 4 (2020): 325–329. 33532708 10.1002/ame2.12142 PMC7824966

[61] C. Wan , J. Shao , S. R. Gilbert , et al., “Role of HIF‐1α in Skeletal Development,” Annals of the New York Academy of Sciences 1192 (2010): 322–326. 20392254 10.1111/j.1749-6632.2009.05238.x PMC3047468

[62] J. You , M. Liu , M. Li , et al., “The Role of HIF‐1α in Bone Regeneration: A New Direction and Challenge in Bone Tissue Engineering,” International Journal of Molecular Sciences 24, no. 9 (2023): 8029. 37175732 10.3390/ijms24098029 PMC10179302

[63] Q. Qin , Y. Liu , Z. Yang , et al., “Hypoxia‐Inducible Factors Signaling in Osteogenesis and Skeletal Repair,” International Journal of Molecular Sciences 23, no. 19 (2022): 11201. 36232501 10.3390/ijms231911201 PMC9569554

[64] Y. Pan , Z. Liu , Y. Tang , et al., “HIF‐1α Drives the Transcription of NOG to Inhibit Osteogenic Differentiation of Periodontal Ligament Stem Cells in Response to Hypoxia,” Experimental Cell Research 419, no. 2 (2022): 113324. 36002046 10.1016/j.yexcr.2022.113324

[65] P. Lertkiatmongkol , D. Liao , H. Mei , Y. Hu , and P. J. Newman , “Endothelial Functions of Platelet/Endothelial Cell Adhesion Molecule‐1 (CD31),” Current Opinion in Hematology 23, no. 3 (2016): 253–259. 27055047 10.1097/MOH.0000000000000239 PMC4986701

[66] J. R. Privratsky , D. K. Newman , and P. J. Newman , “PECAM‐1: Conflicts of Interest in Inflammation,” Life Sciences 87, no. 3/4 (2010): 69–82. 20541560 10.1016/j.lfs.2010.06.001 PMC2917326

[67] Q. Zhao , X. Shen , W. Zhang , G. Zhu , J. Qi , and L. Deng , “Mice With Increased Angiogenesis and Osteogenesis Due to Conditional Activation of HIF Pathway in Osteoblasts Are Protected From Ovariectomy Induced Bone Loss,” Bone 50, no. 3 (2012): 763–770. 22193550 10.1016/j.bone.2011.12.003

[68] S. H. Shomento , C. Wan , X. Cao , et al., “Hypoxia‐Inducible Factors 1α and 2α Exert Both Distinct and Overlapping Functions in Long Bone Development,” Journal of Cellular Biochemistry 109, no. 1 (2010): 196–204. 19899108 10.1002/jcb.22396

[69] H. J. Knowles , “Distinct Roles for the Hypoxia‐Inducible Transcription Factors HIF‐1α and HIF‐2α in Human Osteoclast Formation and Function,” Scientific Reports 10, no. 1 (2020): 21072. 33273561 10.1038/s41598-020-78003-z PMC7713367

[70] X. Meng , B. Wielockx , M. Rauner , and A. Bozec , “Hypoxia‐Inducible Factors Regulate Osteoclasts in Health and Disease,” Frontiers in Cell and Developmental Biology 9 (2021): 658893. 33816509 10.3389/fcell.2021.658893 PMC8014084

[71] R. H. Christenson , “Biochemical Markers of Bone Metabolism: An Overview,” Clinical Biochemistry 30, no. 8 (1997): 573–593. 9455610 10.1016/s0009-9120(97)00113-6

[72] A. Nair , S. C. Chuang , Y. S. Lin , et al., “Characterization of Collagen Response to Bone Fracture Healing Using Polarization‐SHG,” Scientific Reports 12, no. 1 (2022): 18453. 36323698 10.1038/s41598-022-21876-z PMC9630316

[73] M. Tzaphlidou , “Bone Architecture: Collagen Structure and Calcium/Phosphorus Maps,” Journal of Biological Physics 34, no. 1/2 (2008): 39–49. 19669491 10.1007/s10867-008-9115-y PMC2577747

[74] M. Tsioumpekou , N. Papadopoulos , F. Burovic , C. H. Heldin , and J. Lennartsson , “Platelet‐Derived Growth Factor (PDGF)‐Induced Activation of Erk5 Map‐Kinase Is Dependent on Mekk2, Mek1/2, PKC and PI3‐kinase, and Affects BMP Signaling,” Cellular Signalling 28, no. 9 (2016): 1422–1431. 27339033 10.1016/j.cellsig.2016.06.013

[75] S. Almubarak , H. Nethercott , M. Freeberg , et al., “Tissue Engineering Strategies for Promoting Vascularized Bone Regeneration,” Bone 83 (2016): 197–209. 26608518 10.1016/j.bone.2015.11.011 PMC4911893

[76] H. R. Kwon , J. H. Kim , J. P. Woods , and L. E. Olson , “Skeletal Stem Cell Fate Defects Caused by Pdgfrb Activating Mutation,” Development 148, no. 23 (2021): dev199607. 34738614 10.1242/dev.199607 PMC8714075

[77] A. M. Böhm , N. Dirckx , R. J. Tower , et al., “Activation of Skeletal Stem and Progenitor Cells for Bone Regeneration Is Driven by PDGFRβ Signaling,” Developmental Cell 51, no. 2 (2019): 236–254.e12. 31543445 10.1016/j.devcel.2019.08.013

[78] H. R. Kwon , J. H. Kim , J. P. Woods , L. E. Olson , “An Activating Mutation in Causes Skeletal Stem Cell Defects With Osteopenia and Overgrowth in Mice,” bioRxiv 1 (2021): 427619.

[79] G. F. Pierce , T. A. Mustoe , B. W. Altrock , T. F. Deuel , and A. Thomason , “Role of Platelet‐Derived Growth Factor in Wound Healing,” Journal of Cellular Biochemistry 45, no. 4 (1991): 319–326. 10.1002/jcb.240450403 2045423

[80] Z. Gao , T. Sasaoka , T. Fujimori , et al., “Deletion of the PDGFR‐β Gene Affects Key Fibroblast Functions Important for Wound Healing*,” Journal of Biological Chemistry 280, no. 10 (2005): 9375–9389. 15590688 10.1074/jbc.M413081200

[81] S. Onuora , “PDGFR Signalling Implicated in Anti‐Resorptive Effects of Sclerostin Blockade,” Nature Reviews Rheumatology 20, no. 7 (2024): 396–398. 10.1038/s41584-024-01133-5 38839845

[82] C. Thouverey , P. Apostolides , J. Brun , J. Caverzasio , and S. Ferrari , “Sclerostin Blockade Inhibits Bone Resorption Through PDGF Receptor Signaling in Osteoblast Lineage Cells,” JCI Insight 9, no. 10 (2024): e176558. 38713511 10.1172/jci.insight.176558 PMC11141910

[83] M. M. Durdan , R. D. Azaria , and M. M. Weivoda , “Novel Insights Into the Coupling of Osteoclasts and Resorption to Bone Formation,” Seminars in Cell & Developmental Biology 123 (2022): 4–13. 34756783 10.1016/j.semcdb.2021.10.008 PMC8840962

[84] S. C. Moser and B. C. J. van der Eerden, , “Osteocalcin—A Versatile Bone‐Derived Hormone,” Frontiers in Endocrinology 9 (2019): 794. 30687236 10.3389/fendo.2018.00794 PMC6335246

[85] S. C. Manolagas , “Osteocalcin Promotes Bone Mineralization but Is not a Hormone,” PLoS Genetics 16, no. 6 (2020): e1008714. 32484816 10.1371/journal.pgen.1008714 PMC7266291

[86] P. H. Schlesinger , H. C. Blair , D. Beer Stolz , et al., “Cellular and Extracellular Matrix of Bone, With Principles of Synthesis and Dependency of Mineral Deposition on Cell Membrane Transport,” American Journal of Physiology‐Cell Physiology 318, no. 1 (2020): C111–C124. 31532718 10.1152/ajpcell.00120.2019 PMC6985832

[87] I. N. Amirrah , Y. Lokanathan , I. Zulkiflee , M. F. M. R. Wee , A. Motta , and M. B. Fauzi , “A Comprehensive Review on Collagen Type I Development of Biomaterials for Tissue Engineering: From Biosynthesis to Bioscaffold,” Biomedicines 10, no. 9 (2022): 2307. 36140407 10.3390/biomedicines10092307 PMC9496548

[88] M. Zhang , W. Yu , K. Niibe , et al., “The Effects of Platelet‐Derived Growth Factor‐BB on Bone Marrow Stromal Cell‐Mediated Vascularized Bone Regeneration,” Stem Cells International 2018 (2018): 1–16. 10.1155/2018/3272098 PMC6234453 30515221

[89] B. Xue , Y. Li , Z. Fu , H. Ping , and K. Wang , “Intrafibrillar Growth of Hydroxyapatite Nanocrystals in Multiscale Collagen,” Crystals 13, no. 4 (2023): 692.

[90] W. Zhu , J. Kim , C. Cheng , et al., “Noggin Regulation of Bone Morphogenetic Protein (BMP) 2/7 Heterodimer Activity In Vitro,” Bone 39, no. 1 (2006): 61–71. 16488673 10.1016/j.bone.2005.12.018 PMC2943335

[91] A. S. Çakmak , S. Fuerkaiti , D. Karagüzel , Ç. Karaaslan , and M. Gümüşderelioğlu , “Enhanced Osteogenic Potential of Noggin Knockout C2C12 Cells on BMP‐2 Releasing Silk Scaffolds,” ACS Biomaterials Science & Engineering 9, no. 11 (2023): 6175–6185. 37796024 10.1021/acsbiomaterials.3c00506 PMC10646847

[92] P. Aspenberg , C. Jeppsson , and A. N. Economides , “The Bone Morphogenetic Proteins Antagonist Noggin Inhibits Membranous Ossification,” Journal of Bone and Mineral Research 16, no. 3 (2001): 497–500. 11277267 10.1359/jbmr.2001.16.3.497

[93] Z. Xie , P. Wang , Y. Li , et al., “Imbalance Between Bone Morphogenetic Protein 2 and Noggin Induces Abnormal Osteogenic Differentiation of Mesenchymal Stem Cells in Ankylosing Spondylitis,” Arthritis & Rheumatology 68, no. 2 (2016): 430–440. 26413886 10.1002/art.39433

[94] J. A. McMahon , S. Takada , L. B. Zimmerman , C. M. Fan , R. M. Harland , and A. P. McMahon , “Noggin‐Mediated Antagonism of BMP Signaling Is Required for Growth and Patterning of the Neural Tube and Somite,” Genes & Development 12, no. 10 (1998): 1438–1452. 9585504 10.1101/gad.12.10.1438 PMC316831

[95] Z. Jiang , G. Liang , Y. Xiao , et al., “Targeting the SLIT/ROBO Pathway in Tumor Progression: Molecular Mechanisms and Therapeutic Perspectives,” Therapeutic Advances in Medical Oncology 11 (2019): 1758835919855238. 31217826 10.1177/1758835919855238 PMC6557020

[96] M. Tong , T. Jun , Y. Nie , J. Hao , and D. Fan , “The Role of the SLIT/ROBO Signaling Pathway,” Journal of Cancer 10, no. 12 (2019): 2694–2705. 31258778 10.7150/jca.31877 PMC6584916

